# Diphtheria

**Published:** 1891-04

**Authors:** R. J. Mitchell

**Affiliations:** Girard, Ill.


### DIPHTHERIA.

BY R. J. MITCHELL, M. D., GIRARD, ILL.

As it is my belief that this disease is primarily always located in the pharyngeal cavity, and that the symptoms are mild and the deposit limited in area in its first onset, and that the nasal and laryngeal varieties and the so-called malignant form of the disease are only later phases of its development and the natural result of the septic condition existing in the faucial region acting through the medium of the circulation and respiration, I therefore divide the disease into two stages, primary and secondary. The first, or primary stage, dates from the beginning of the first symtoms, viz.: fever, anorexia, sore throat, swelling of the cervical glands, together with the characteristic patches in the throat, when, if the case has made no progress towards recovery, a new formation of deposit is thrown out upon fresh surfaces, which extends either upwards into the nasal cavity and covers the palate and roof of the mouth, or downwards into the larynx and produces croup. It is the first stage of the disease that is important to the physiaian in point of treatment, while the second stage, as every one knows by experience, offers but little to hope for in this respect. I therefore emphasize the importance of early diagnosis before the system has become too much affected by the poison, as the disease in its incipiency is, I believe, strictly local, so that by early and energetic treatment, with properly selected remedies, it can be controlled and its mortality greatly reduced.

Diphtheria is, according to the latest research and generally accepted opinion, dependent up a specific germ from its origin, known as Loeffler's bacillus, which finds lodgment on or in the mucous surface of the pharynx, and does not, as we are told, enter the circulation, but elaborates a ptomaine or poisonous product that does. It would, therefore, from a theoretical point of view, be but a comparatively easy matter to destroy this death-dealing organism with some of our numerous never-failing germicides; but practice does not prove what theory suggests for treatment of this disease in this case, as in some others, and we have been doomed to disappointment, for the whole list of parasiticides has been well nigh exhausted in searching for a sure remedy. Many of the poisonous drugs have been administered internally as well as locally, and some of the most active of these germ-destroyers have yielded the poorest results, for the reason that if they be used in strength sufficient to destroy the microbe, situated in an open avenue to the stomach and protected by a fortification of its own building, the exudate would poison the patient, and I am led to believe that the best results in the treatment of this disease will never be obtained from this class of remedies, and take pleasure in attempting to prove the usefulness of a harmless treatment which I have employed for the past 11 years exclusively with satisfaction, which consists of the following:

Quinine, gr. xxx.
Hydrochloric acid, gtt. xxx.
Water, oz. j.

To be applied with an atomizer to the patches in the throat every two hours, using three or four compressions of the bulb, taking pains to expose the diseased parts thoroughly and allowing no drinks to be taken for one-half hour afterwards. Quinine, grs.xx: tincture iron, ʒiij; aquae ad. ℥ ij; teaspoonful every two hours, followed by alcohol diluted to 1-6 or whisky diluted to 1-3, to wash it down. The use of tincture of iron and alchohol for this purpose seems to have met with general favor, while the use of quinia locally as an antiseptic has been quite limited, though as far as I can learn it has yielded good results to all who have thus employed it. Professor Rochester has been referred to as authority for the local use of this drug in this disease. He employed it by insufflation, two grains every two hours being blown into the throat, with good results. Dr. Cleveland speaks favorably of its use in this way. Dr. Burghardt, in the *Vienna Medicinische Wochenschrift*, of October 5, 1889, speaks highly of the use of quinine and sulphur, equal parts, by insufflation, in the treatment of diphtheria, three grains being blown into the diseased parts after cleansing them with a gargle or drink of water, making application but twice a day, and recommends this treatment as being especially adapted to young children, on account of ease of application. He allows nothing of the nature of food or drink to be taken for an hour afterwards, and further states that since 1882 he has treated in this way thirty-three cases in patients from one to twenty-four years of age, without a fatal result; whereas, by other methods, he has lost almost three-fourths of his diphtheria patients. Between the insufflations he gives tincture of iron, wine and brandy as tonics, and if there is much blood poisoning, he gives large doses of iron, ergot, lemon juice, etc. All of his cases were seen early in the attack. He was led to adopt this treatment from observing the effects of sulphur on diseased vines in destroying the fungus oidum vitus, and knowing the germicidal as well as the tonic properties of quinine, the happy thought struck him to combine them for this purpose, and the combination seems to be a good one, as the results show; but to which element of this combination the results are due, he does not explain, nor will I attempt to say, further than that for me, quinine without the sulphur has done as well, as my results also will show. In support of this treatment, I submit the following cases, all of which were followed by the characteristic sequela, viz., paralysis.

CASE I.—Ethel D., aged 6, was seen April 15, 1889. She had been suffering a day or two with what was supposed to be a bad cold, having fever, sore throat, etc. Examination showed throat inflamed, no patches on the tonsils, severe swelling of the cervical glands and painful deglutition; but on my return the next day patches were found on the tonsils. She was put on the quinine spray every two hours; solution of quinine and iron every two hours, with the diluted whisky, for five days, when the throat cleared, and the solution of iron and quinine was then continued until convalescence was established. About three weeks after, she was brought to my office on account of difficulty in swallowing liquids and in speaking so as to be understood. As her appetite was good and general condition improving, she received no further treatment, and recovered from the paralysis in about six weeks.

CASE II.—Lucy McL., aged 10, was taken with high fever and severe sore throat the day before I saw her, while visiting in a neighboring town, and when I saw her, August 23rd, she had fever, painful deglutition, swelling in the angles of the jaws. Examination of the throat showed a thick, white patch of quadrilateral shape located on the anterior aspect of the tonsils and uvula, extending upwards to the edge of the palate, binding them together in a solid mass, obscuring them entirely from view. She was put upon the treatment immediately, and as she could expose her throat well without a tongue-depressor, being old enough to fully realize her dangerous condition and the importance of treatment, she got the full benefit of the spray, together with the iron and alcohol· There was no change until the fourth day of the treatment except the subsidence of the fever, when the thick patch of membrane loosened at its upper edge and hung forward like a mirror from a wall, vibrating with the respiration, which was made through the mouth. I made slight efforts to remove the partially detached membrane, and finding it impractical to do so, left it, and on my return next day I found it gone, but replaced by a new and much thinner formation, which, under the continued use of the spray, disappeared thirty-six hours later, and the throat became clear. The case progressed favorably under the quinine and iron solution. Abont three weeks after my last visit she called at my office, complaining that she could not see to read, and on trial I found that she could barely read the large letters from the heading of a newspaper. I prescribed a pair of No. 7 convex glasses to correct the error of refraction. The power of accommodation gradually returned, so that at the end of six weeks vision was apparently normal.

CASE III.—Johnny McL. was taken about a week after his sister Lucy, and as the parents had become expert in the application of the treatment on the girl, I was not called until the boy had been under treatment about three days after the attack. I found the characteristic symptoms present, or had been, fever subsiding. On inspection of the throat, a deposit was seen, limited to the uvula, beginning at its base, covering its full width, extending downwards, its free or detached lower end of band or ribbon shaped reaching the vocal cords, as was evidenced by the complete absence of phonation. The tonsils and other parts of the faucial and oral cavity were clean and red. Treatment continued until the seventh day, when the throat cleared up and the voice immediately returned. Paralysis of the faucial muscles made its appearance two weeks later, which lasted about six weeks. He was very weak when convalescence began, and made a slow but complete recovery.

CASE IV.—M. W., aged 7, the second of three cases in the family, August 7, 1889. Symptoms regular; fever, sore throat, etc.; deposit confined to the tonsils. Treatment began early. She could not take the spray successfully on account of inability to bear the use of the tongue depressor, which caused her to retch and vomit her food, and I had, therefore, to rely upon the quinine iron solution, which I gave every two hours, with plenty of whisky, till the tenth day, when the deposit disappeared, and afterwards three times a day till convalescence was established. Her throat muscles became paralyzed, so that it was next to impossible for her to swallow liquids, and it was very difficult to understand her when speaking. The external rectus of one of the eyes became affected, and strabismus prevailed for a month. She was obliged to stay out of school for three months on account of her inability to speak distinctly.

In all of these cases except the first the urine was examined and found to be albuminous. I will not give in detail the history of the other seven cases, for I think from those reported physicians can fully understand their nature, as well as the nature of the epidemic prevailing throughout the country at that time, of which they form a part. These patients range in age from three to fourteen years. None of them were followed by sequelae, but were, nevertheless, well marked cases of diphtheria. Two of these cases, aged three and fourteen respectively, were quite severe, the deposits on the tonsils being extensive, together with decided swelling of the cervical glands and albuminous urine. The eleven cases included in this report occurred between the dates of April 15th and September 16th, 1889, as follows: One case in April, one in May, eight in August and one in September.

It affords me pleasure to report the successful management of these eleven cases which it has been my good fortune to see recover under the local use of quinine, aided by tincture of iron and alcohol, to which I will add twenty-one cases reported last year, giving me a total of thirty-two cases treated by this method without a single fatal result, against a mortality of over 70 per cent. of the cases treated by me by other methods, and I am therefore forced to conclude that this mode of treatment will compare favorably with any other, and is deseiving of a more general recognition and further trial.

---

ERGOT IN OBSTETRICAL PRACTICE—ITS USE AND ABUSE.—Edwin B. Shaw (*Kansas Medical Journal*, December, 1890) gives the following summary: No thoughtful practitioner would ever use it in a case of retarded placenta.

1. It stands without a rival as a prophylactic against *post partum* hemorrhage.

2. It acts as a hemostatic in nature's way, and is both safe and reliable.

3. It should be given just before or immediately after the birth of the head, so as to obtain its action when needed—soon after the emptying of the uterine contents.

4. That it promotes the process of involution, is rationally explained by the fact that it produces contraction and retraction of uterine muscular fibre, and contraction of the arterioles, thereby favoring fatty degeneration by diminishing blood supply.

5. It diminishes and prevents after-pains, adding greatly to the comfort of the patient.

6. It lessens, in some way, the dangers of septic complications.

7. It shortens the stage of convalescence.

8. Obstetricians of large experience, such as Goodell and Parvin, of Philadelphia; and Busey, of Washington, testify that they have never observed any bad results from the judicious use of ergot.

---

HARRASSING COUGH.—

℞ Mist. glycyrrhiz. Co., ℥ ij.
Sp. chloroformi, ʒ ss.
Sp. ammonii aromat., ℥ j.

M. Sig. A teaspoonful every two hours.

—*Dr. Beverly Robinson.*

